# Propagation of Tau aggregates

**DOI:** 10.1186/s13041-017-0298-7

**Published:** 2017-05-30

**Authors:** Michel Goedert, Maria Grazia Spillantini

**Affiliations:** 1 MRC Laboratory of Molecular Biology, Francis Crick Avenue, Cambridge, CB2 0QH UK; 2 Department of Clinical Neurosciences, Clifford Allbutt Building, University of Cambridge, Hills Road, Cambridge, CB2 0AH UK

**Keywords:** Alzheimer’s disease, Amyloid, Cell-to-cell spreading, Disease propagation, Prion-like, Protein strains, Tau, Tauopathies

## Abstract

Since 2009, evidence has accumulated to suggest that Tau aggregates form first in a small number of brain cells, from where they propagate to other regions, resulting in neurodegeneration and disease. Propagation of Tau aggregates is often called prion-like, which refers to the capacity of an assembled protein to induce the same abnormal conformation in a protein of the same kind, initiating a self-amplifying cascade. In addition, prion-like encompasses the release of protein aggregates from brain cells and their uptake by neighbouring cells. In mice, the intracerebral injection of Tau inclusions induced the ordered assembly of monomeric Tau, followed by its spreading to distant brain regions. Short fibrils constituted the major species of seed-competent Tau. The existence of several human Tauopathies with distinct fibril morphologies has led to the suggestion that different molecular conformers (or strains) of aggregated Tau exist.

## Introduction

Common human neurodegenerative diseases are characterized by the presence of abundant ordered assemblies with the properties of amyloid fibrils [1, 2]. These structures are typically unbranched and 10–20 nm in diameter; they are composed of β-strands running perpendicular to the fibre axis and β-sheets running parallel to the long axis of the fibrils. Each inclusion has a single protein as its major component, with Aβ, Tau and α-Synuclein being the most commonly involved. These proteins undergo a transformation from a soluble to an insoluble filamentous state, with a number of intermediates. The majority of neurodegenerative diseases is defined by one type of inclusion. Alzheimer’s disease (AD), the most prevalent, is characterized by two types of abundant inclusions, Aβ deposits and Tau assemblies.

Most cases of disease are sporadic, but a small percentage is inherited, often in a dominant manner. Inherited cases are caused by mutations in the genes encoding the proteins that make up the inclusions, or proteins that increase their production. Mutations in *MAPT*, the Tau gene, give rise to an inherited form of frontotemporal dementia and parkinsonism with abundant filamentous Tau inclusions in brain [3].

From genetic data, it is known that a dysfunction of the amyloid precursor protein (APP) initiates the disease process in familial AD [4]. The same may be true of sporadic AD. However, it is also clear that pathological changes in Tau correlate better with nerve cell dysfunction than Aβ deposits [5, 6]. Moreover, a close relationship between tau inclusions and nerve cell loss is well established in hippocampus and cerebral cortex [7]. Furthermore, it remains to be shown how changes in APP can cause neurotoxicity related to Tau aggregation.

For many years, cell autonomous mechanisms were believed to account for human neurodegenerative diseases, implying that the same aggregation events occur in brain cells, independently of neighbouring cells, resulting in degeneration. In *post mortem* brains, protein inclusions are present in thousands of cells. An alternative view is that the first inclusions form in a small number of cells, from where they propagate to normal cells through non-cell autonomous mechanisms and well-defined pathways depending on the underlying disease, resulting in degeneration.

Propagation of pathology is commonly called “prion-like”, referring to the intercellular spreading of protein aggregates. The acronym “prion” stands for “proteinaceous infectious particle”, reflecting intercellular propagation and interorganismal transmission [8]. There is no evidence to suggest that Tauopathies can transfer between humans, hence the use of “prion-like.” Propagation of aggregates requires their release into the extracellular space, uptake by connected cells and seeded aggregation of soluble proteins. Studying the underlying mechanisms may lead to the identification of novel therapeutic targets.

## TAU isoforms

**Fig. 1:**
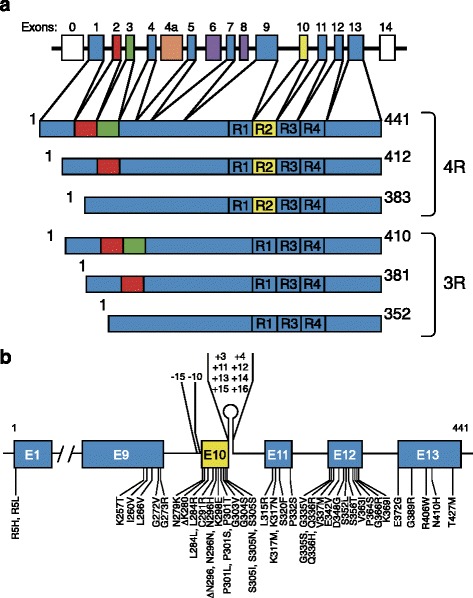
Human brain Tau isoforms and *MAPT* mutations. **a** *MAPT* and the six Tau isoforms expressed in adult human brain. *MAPT* consists of 16 exons (E). Alternative mRNA splicing of E2 (*red*), E3 (*green*), and E10 (*yellow*) gives rise to six tau isoforms (amino acids 352–441). Constitutively spliced exons (E1, E4, E5, E7, E9, E11, E12, E13) are shown in *blue*. E0, which is part of the promoter, and E14 are noncoding (*white*). E6 and E8 (*violet*) are not transcribed in human brain. E4a (*orange*) is only expressed in the peripheral nervous system. The repeats (R1-R4) are shown, with three isoforms having four repeats each (4R) and three isoforms having three repeats each (3R). Each repeat is 31 amino acids in length. Exons and introns are not drawn to scale. **b** Mutations in *MAPT* in cases of frontotemporal dementia and parkinsonism linked to chromosome 17 (FTDP-17 T); 49 coding region mutations and 10 intronic mutations flanking E10 are shown

Six Tau isoforms are expressed in adult human brain (Fig. 1a) [9]. They range from 352 to 441 amino acids and are produced by alternative mRNA splicing of transcripts from *MAPT*. The six isoforms are natively unfolded and differ by the presence or absence of inserts of 29 or 58 amino acids in the amino-terminal half, and the inclusion or not, of the 31 amino acid repeat encoded by exon 10 of *MAPT* in the carboxy-terminal half. Inclusion of exon 10 results in the production of three Tau isoforms with four repeats each (4R), and its exclusion in a further three isoforms with three repeats each (3R). Big Tau, which carries an additional large exon in the amino-terminal half, is expressed in the peripheral nervous system [10, 11]. Together with some adjoining sequences, the repeats constitute the microtubule-binding domains of Tau [12].

The dwell time of Tau on individual microtubules is short and not significantly different between isoforms [13, 14]. Tau promoted microtubule assembly in the processes of differentiated PC12 cells, but it is not clear if it also stabilised microtubules. Its short dwell time on microtubules makes it possible for Tau to interact with additional molecules, such as actin and protein phosphatase 2A. In brain, Tau is subject to a number of post-translational modifications, including phosphorylation, acetylation, methylation, glycation, isomerisation, O-GlcNAcylation, nitration, sumoylation, ubiquitination and truncation [15].

Similar amounts of 3R and 4R Tau are expressed in the cerebral cortex of adults [16]. In developing human brain, only the shortest Tau isoform is present. 3R, 4R and 5R Tau isoforms are found in the brains of adult chickens [17], whereas most adult rodents express only 4R Tau. What is conserved between species is the expression of one hyperphosphorylated 3R Tau isoform lacking amino-terminal inserts during vertebrate development. Similar repeats are present in the high-molecular weight proteins MAP2 and MAP4 [18, 19]. It has been suggested that MAP4 derives from a non-vertebrate ancestor, whereas MAP2 and Tau may have shared a more recent common ancestor [20]. The genomes of *Caenorhabditis elegans* and *Drosophila melanogaster* each encode one protein with Tau-like repeats [21, 22].

## TAU assembly

Full-length Tau assembles into filaments through some of its repeats and adjoining sequences, with the amino-terminal half and most the carboxy-terminus forming the fuzzy coat [23–27]. Tau filaments from AD brain and those assembled from expressed protein have a cross-β structure characteristic of amyloid fibrils [28], with their cores consisting of approximately 90 amino acids. The core of Tau filaments overlaps with part of the region that binds to microtubules, implying that pathological assembly and physiological function are mutually exclusive.

Phosphorylation of Tau negatively regulates its ability to interact with microtubules and filamentous Tau is abnormally hyperphosphorylated [29]. It remains to be seen if phosphorylation is a trigger for aggregation. Many publications equate Tau phosphorylation with aggregation. This may not be correct. It has for instance been shown that highly phosphorylated Tau forms in a reversible fashion during hibernation [30].

In AD, chronic traumatic encephalopathy (CTE), post-encephalitic parkinsonism and several other Tauopathies, all six isoforms are present in the disease filaments [1]. In AD, filaments are either paired helical or straight, with both types sharing a common structural subunit [31]. In other diseases-such as progressive supranuclear palsy (PSP), corticobasal degeneration (CBD), argyrophilic grain disease (AGD), globular glial Tauopathy (GGT) and aging-related Tau astrogliopathy (ARTAG)-only isoforms with 4R Tau are found in the filaments [1]. In Pick’s disease (PiD), 3R Tau isoforms predominate in the inclusions [1]. Unlike AD, these diseases lack Aβ deposits. The morphologies of Tau filaments in different diseases vary, even when they are made of the same isoforms.

It has been suggested that patients with AD-type neurofibrillary degeneration restricted to medial temporal lobe and hippocampus, who lack Aβ deposits, suffer from “primary age-related Tauopathy” (PART), a condition that differs from AD [32]. It has also been proposed that tangle-only dementia (TD), a relatively rare form of dementia, represents a severe form of PART. However, the view that PART is different from AD has been challenged, because it is clinically and neuropathologically similar to what appear to be the early pathological stages of AD [33].

The interaction in vitro between unphosphorylated, full-length, recombinant Tau and some negatively charged compounds, such as sulphated glycosaminoglycans, results in filament assembly [34, 35]. Filaments are decorated by antibodies directed against the amino-and carboxy-termini of Tau, but not by an antibody directed against the repeats. These findings, which indicate that the repeat region is inaccessible to the antibody, are identical to those obtained in AD and other human Tauopathies [36]. However, the mechanisms leading to filament formation inside human brain cells in sporadic Tauopathies remain to be identified. Heparin is probably not involved.

Hexapeptide sequences in R2 (amino acids 275–280, VQIINK) and R3 (amino acids 306–311, VQIVYK) are essential for Tau filament assembly [37, 38]. Microcrystals of a peptide comprising residues 306–311 formed steric zippers [39]. In complex with microtubules, these hexapeptide sequences are in a hairpin conformation [40], consistent with the view that microtubule binding and pathological assembly of Tau are mutually exclusive. Residues 310–313 in Tau (YKPV) differ from the equivalent residues in MAP2 (TKKI). When the latter were changed to YKPV, MAP2c also assembled into filaments [41]. The longest human brain Tau isoform (2N4R, 441 amino acids) contains cysteines in R2 (residue 291) and R3 (residue 322). Intra-and intermolecular disulphide bonds may play a role in the assembly of 3R and 4R Tau into filaments.

## Genetics of *MAPT*

The link between Tau dysfunction and neurodegeneration was established through human genetics. In June 1998, mutations in *MAPT* were reported in a dominantly inherited form of frontotemporal dementia and parkinsonism linked to chromosome 17q21-22 [42–44]. Fifty-nine pathogenic *MAPT* mutations had been identified by January 2017 (Fig. 1b). Behavioural symptoms are the most common clinical sign. However, language disturbances are sometimes seen, as are signs of parkinsonism and motor neuron disease. The ages of onset are variable, but can be as early as in the third decade. *MAPT* mutations are always associated with abundant Tau inclusions in nerve cells or in both nerve cells and glial cells [3]. By electron microscopy, Tau filament morphologies vary [45].

Mutations in *MAPT* account for around 5% of cases of FTD. They are concentrated in exons 9–12 (encoding R1-R4) and the introns flanking exon 10, and can be divided into those with a primary effect at the protein level and those affecting the alternative splicing of Tau pre-mRNA (Fig. 1). Mutations that act at the protein level change or delete single amino acids, reducing the ability of Tau to interact with microtubules. Some mutations also promote the assembly of Tau into filaments. Mutations with a primary effect at the RNA level are intronic or exonic and increase the alternative mRNA splicing of exon 10 of *MAPT*. This affects the ratio of 3R to 4R isoforms, resulting in the relative overproduction of 4R Tau, and its assembly into filaments. In neurons derived from induced pluripotent stem cells of patients with intronic *MAPT* mutations, 4R Tau was expressed during development, unlike what happens normally [46, 47].

Aggregated Tau can show different isoform patterns, depending on the *MAPT* mutations [48]. Mutations V337M in exon 12 and R406W in exon 13 give rise to insoluble Tau bands of 60, 64 and 68 kDa and a weaker band of 72 kDa. Following dephosphorylation, six bands are present that align with recombinant Tau, like what is seen in AD. The brains of many individuals with *MAPT* mutations in exons 9–13 (K257T, L266V, ΔK280, S305N, G272V, L315R, S320F, S320Y, P332S, Q336H, Q336R, K369I, E372G and G389R) are characterized by abundant Pick bodies made predominantly of 3R Tau. As in sporadic PiD, insoluble Tau shows strong bands of 60 and 64 kDa. However, in most cases, variable amounts of the 68 and 72 kDa bands are also observed. A third pattern is characteristic of *MAPT* mutations that affect the alternative mRNA splicing of exon 10 (intronic mutations and exonic mutations N279K, L284L, L284R, ΔN296, N296D, N296H, N296N, S305I, S305N and S305S). Insoluble Tau runs as two strong bands of 64 and 68 kDa and a weaker band of 72 kDa; following dephosphorylation, three bands are present that align with recombinant 4R Tau (isoforms of 383, 412 and 441 amino acids). A similar pattern of pathological Tau bands is observed for mutations in exon 10, such as P301L, which have their primary effects at the protein level. Aggregation of 4R Tau has also been described for mutations I260V in exon 9, K317N in exon 11, E342V in exon 12 and N410H in exon 13, showing that it is possible to alter 3R and 4R Tau mRNAs through mutations located outside exon 10.

In populations of European descent, *MAPT* is characterised by two haplotypes resulting from a 900 kb inversion (H1) or non-inversion (H2) polymorphism [49]. Inheritance of H1 is a risk factor for PSP, CBD and Parkinson’s disease (PD) [48]. This has been confirmed in genome-wide association studies. The association with PD is particularly surprising, since PD is not characterised by the presence of Tau inclusions.

For PSP and CBD, an association has also been found with an allele at the MOBP/SLC25A38 locus, which results in elevated levels of appoptosin, a protein that activates caspase-3, which can cleave Tau [50]. This may result in increased aggregation of 4R Tau. The association of H1 with PSP had a higher odds ratio than that between APOEε4/ε4 and AD [51]. APOEε4 is the major risk factor allele for late-onset AD [52]. H2 is associated with increased expression of exon 3 of *MAPT* in grey matter, suggesting that inclusion of exon 3 is protective [53]. Reduced expression of 1N4R has also been associated with H2 [54]. Tau isoforms containing exons 2 and 10 promote aggregation, whereas exon 3-containing isoforms are inhibitory [55].

Even though all six Tau isoforms form paired helical and straight filaments, there are no known mutations in *MAPT* that give rise to AD. Tau with an A152T substitution has been reported to be a risk factor for AD, as well as for PSP, CBD and unusual Tauopathies ([56–58], but see also [59]).

## Propagation of TAU aggregates

**Fig. 2:**
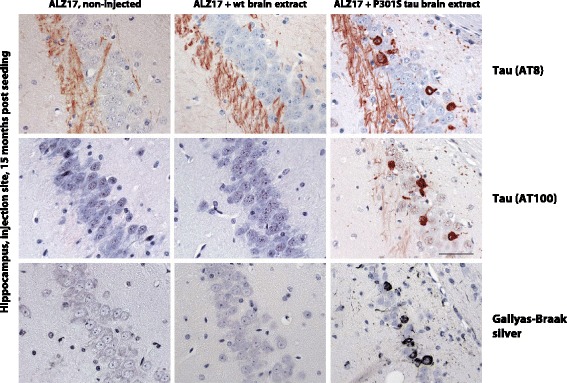
Induction of filamentous Tau pathology in mice transgenic for wild-type human Tau (line ALZ17) following injection with brain extracts from symptomatic mice transgenic for human mutant P301S tau. Staining of the hippocampal CA3 region of 18-month-old ALZ17 mice with anti-Tau antibodies AT8 and AT100 and Gallyas-Braak silver. Non-injected (*left*), 15 months after injection of brain extract from non-transgenic control mice (*middle*) and 15 months after injection with brain extract from 6-month-old mice transgenic for human P301S Tau (*right*). The sections were counterstained with haematoxylin. Scale bar = 50 μm

Tau assembly follows a nucleation-elongation mechanism. Experimentally, nucleation can be circumvented by external seeds of preformed Tau filaments. In 2009, prion-like mechanisms were first implicated in Tau pathology (Fig. 2) [60, 61]. Since then, evidence has accumulated to suggest that Tau assemblies, when applied extracellularly, can “seed” the formation of aggregates, followed by their spreading to other cells. Because Tau is an intracellular protein, its propagation requires seeding, as well as aggregate uptake and release. Even though monomeric Tau is taken up by cells, from which it can be released, it is probably not able to seed aggregation. Expressed 4R Tau cannot be seeded when it lacks residues 275–280 and 306–311 [62]. Aggregation inhibitors may thus be able to reduce Tau-induced seeding and spreading.

Uptake of ordered Tau assemblies depends on heparan sulphate proteoglycans at the cell surface and may occur through macropinocytosis, at least in cultured cells [63]. The seeds probably escape from endosomal vesicles and induce the assembly of cytoplasmic Tau. Following assembly, Tau aggregates are released from cells through ill-defined mechanisms. Intracellular Tau could transfer between cells through tunnelling nanotubes. Alternatively, it could be released into the extracellular space, either freely or inside vesicles. Antibodies may be able to target extracellular Tau aggregates, provided they are not transported in vesicles. Passive immunisation with anti-Tau antibodies has been shown to reduce the amounts of aggregated, hyperphosphorylated Tau in transgenic mice [64–66]. Similarly, antisense oligonucleotides reduced Tau pathology [67]. The aggregation of Tau is known to be concentration-dependent. Tau assemblies can enter cells, where they are detected and neutralised via a danger response mediated by bound anti-Tau antibodies and the cytosolic Fc receptor tripartite motif protein 21 (TRIM21) [68].

Microglial cells have been reported to promote Tau propagation through exosome-dependent mechanisms [69]. A separate study concluded that Tau was released from cells through an exosome-independent pathway that required heat shock cognate 70, its co-chaperone DnaJ and synaptosomal-associated protein 23 [70]. This work probably described the release of aggregation-incompetent soluble tau. By contrast, optogenetic and chemogenetic approaches have shown that an increase in neural activity can accelerate Tauopathy in transgenic mice [71].

Tau seeds do not need to be phosphorylated, even though seeded Tau aggregates are hyperphosphorylated [62]. Tau assemblies may grow by incorporating unphosphorylated Tau which then undergoes a conformational change and becomes hyperphosphorylated. It remains to be seen if phosphorylation of Tau can influence seeded aggregation.

The intracerebral injection of brain extracts from mice expressing human P301S Tau with inclusions into mice transgenic for wild-type human Tau lacking inclusions (line ALZ17) induced the assembly of wild-type Tau into filaments and its spreading to distant brain regions [60]. No inclusions formed when Tau was depleted from the extracts prior to injection. Aggregated recombinant human Tau also induced inclusion formation, but with a lower efficiency than aggregated Tau from transgenic mouse brain [72, 73]. Similar differences have been described for prions, Aβ, α-synuclein and reactive serum amyloid A assemblies. Recombinant Tau aggregates were more resistant to disaggregation by guanidine hydrochloride and digestion by proteinase K than Tau aggregates from transgenic mouse brain, consistent with the view that more stable aggregates possess lower seeding activity [62].

Distinct conformations of assembled Tau accounted for differences in seeding potency. Tau filaments formed from recombinant P301S Tau following seeding with aggregated Tau from transgenic mouse brain (in the absence of heparin) showed resistance to guanidine hydrochloride, which was similar to that of Tau seeds from the brains of mice transgenic for human P301S Tau. The seeding potency of Tau filaments was like that of brain-derived aggregated Tau.

When presymptomatic P301S Tau transgenic mice were injected intracerebrally with brain extracts from symptomatic animals, Tau inclusions formed rapidly at the injection sites [74]. Contralateral and caudo-rostral propagation was evident in nuclei with strong afferent and efferent connections to the injection sites, indicating that the spread of pathology was dependent on connectivity, not proximity.

The intraperitoneal injection of brain extracts from symptomatic P301S Tau transgenic mice into presymptomatic mice promoted the formation of cerebral Tau inclusions [75]. Aggregated Tau can thus promote inclusion formation in the central nervous system of transgenic mice following peripheral administration. Similar findings have been reported for prions, assembled Aβ and assembled α-synuclein.

**Fig. 3:**
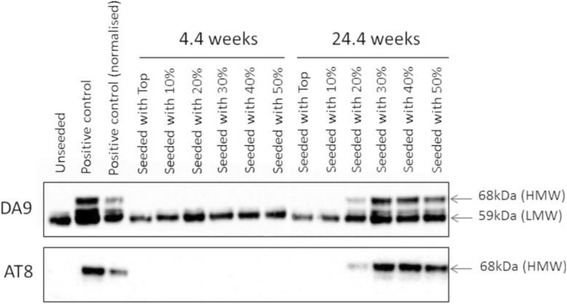
Seeding of Tau aggregation with sucrose gradient fractions from the brains of mice transgenic for human mutant P301S Tau in a cell-based assay. The mice were aged 4.4 weeks (no symptoms, no Tau filaments) or 24.4 weeks (symptoms, abundant Tau filaments). Sucrose gradient fractions were used to seed aggregation of Tau in HEK cells expressing 1N4R Tau with the P301S mutation. The pellet from a 100,000 g spin of seeded cells was analysed by Western blotting for total Tau and Tau phosphorylated at S202/T205 (anti-Tau antibodies DA9 and AT8). Filamentous Tau runs at approximately 68 kDa (HMW, high-molecular weight); non-filamentous Tau runs at approximately 59 kDa (LMW, low-molecular weight). The positive control consisted in seeding with sarkosyl-extracted Tau from unfractionated brains of symptomatic transgenic P301S Tau mice and the normalised positive control was seeding with sarkosyl-extracted Tau from symptomatic mice, normalised for total Tau levels relative to those of the sucrose gradient fractions. Seeding ability correlated with the presence of the 64 kDa band in 24.4-week-old mice (20–50% sucrose gradient fractions). No seeding was observed upon addition of sucrose gradient fractions from the brains of 4.4-week-old mice

We dissected the molecular characteristics of seed-competent Tau from the brains of symptomatic P301S Tau transgenic mice (Fig. 3) [76]. Sucrose gradient fractions caused aggregation in transfected cells only when large Tau aggregates (>10mers) were present. The same fractions induced the formation and spreading of filamentous Tau in presymptomatic transgenic mice, whereas fractions containing monomers and small Tau aggregates were inactive. By immunoelectron microscopy, seed-competent sucrose gradient fractions contained aggregated Tau species ranging from ring-like structures to small fibrils.

## Strains of aggregated TAU

In 2013, we showed that the intracerebral injection of brain homogenates from humans with pathologically confirmed Tauopathies led to the formation of neuronal and glial Tau inclusions in ALZ17 mice [77]. Inclusions formed after inoculation of brain homogenates from all cases of AD, TD, PiD, AGD, PSP and CBD. Brain homogenates from patients with AGD, PSP and CBD produced lesions similar to those of the human disorders. With the exception of PiD (3R), the inclusions of the Tauopathies used were made of either 4R Tau (AGD, PSP and CBD) or a mixture of 3R and 4R Tau (AD and TD).

Injection of PSP homogenates into ALZ17 mice gave rise to silver-positive neuronal and glial Tau aggregates; the latter were similar to tufted astrocytes, the hallmark lesion of PSP. The injection of CBD homogenates produced neuronal inclusions and silver-positive structures reminiscent of astrocytic plaques. With AGD homogenates, argyrophilic grains and silver-negative astrocytic Tau inclusions were seen, like in the human disease. With the exception of PiD, Tau inclusions propagated over time to connected brain regions. Similar inclusions, but fewer in number, formed after the intracerebral injection into non-transgenic mice of brain homogenates from human Tauopathies. Similar findings have been reported by others following the injection of AD brain extracts into wild-type mice [78, 79].

These findings were complemented by studies in HEK293 cells that had been stably transfected with part of 4R Tau (residues 244–372) with mutations P301L and V337M fused to YFP [80]. When exposed to Tau seeds, mutant 4R Tau aggregated. Two Tau strains were isolated, based on distinct inclusion morphologies, as well as different biochemical and structural characteristics. Inoculation of these assemblies into the hippocampus of young mice transgenic for human mutant P301S tau (line PS19) induced Tau pathologies that were stable through serial transmission. When HEK cells expressing mutant 4R Tau were seeded with homogenates from these brains, inclusions formed that were identical to those present initially. In subsequent work, another 18 Tau strains were isolated [81]. It remains to be seen how these strains relate to the molecular conformers of aggregated Tau that are characteristic of human Tauopathies.

Induced Tau pathology propagated serially, when brain homogenates from ALZ17 mice that had received bilateral injections of brain extracts from human P301S Tau transgenic mice 18 months earlier, were injected into 3-month-old ALZ17 mice [77]. In a different experiment, homogenates were prepared from the brains of non-transgenic mice that had been injected bilaterally with AGD brain homogenates 18 months earlier. Twelve months after the intracerebral injection of these homogenates into ALZ17 mice, many neuropil threads and Tau aggregates were present at the injection sites.

Overexpression of full-length human wild-type 2N3R tau or mutant P301S tau resulted in seeded aggregation upon exposure to AD seeds [68, 78]. Similar findings were obtained when primary neurons from wild-type mice were treated with Tau filaments from AD brain [79]. Seeded aggregation of mutant 4R Tau (244–372) was also observed upon exposure to CTE, PSP, CBD and AGD brain extracts [82]. However, these findings were at the light microscopic level. The resulting Tau filament morphologies are not known. Different strains of aggregated Tau may well exist, but additional work is required. In particular, it will be important to determine if Tau strains from human brain possess unique structural features.

## Conclusion

The ordered assembly of Tau represents the gain of toxic function that causes human Tauopathies. Downstream of assembly, Tau propagation and neurodegeneration take place. Small Tau fibrils are the major species responsible for propagation. The molecular Tau species responsible for neurodegeneration remain to be identified.
